# Plasma Kallikrein-Activated TGF-β Is Prognostic for Poor Overall Survival in Patients with Pancreatic Ductal Adenocarcinoma and Associates with Increased Fibrogenesis

**DOI:** 10.3390/biom12091315

**Published:** 2022-09-17

**Authors:** Rasmus S. Pedersen, Neel I. Nissen, Christina Jensen, Jeppe Thorlacius-Ussing, Tina Manon-Jensen, Majken L. Olesen, Lasse L. Langholm, Hadi M. H. Diab, Lars N. Jorgensen, Carsten P. Hansen, Inna M. Chen, Julia S. Johansen, Morten A. Karsdal, Nicholas Willumsen

**Affiliations:** 1 Nordic Bioscience, 2730 Herlev, Denmark; 2 Department of Biomedical Sciences, University of Copenhagen, 2200 Copenhagen, Denmark; 3 Digestive Disease Center, Bispebjerg and Frederiksberg Hospital, University of Copenhagen, 2400 Copenhagen, Denmark; 4 Department of Surgery, Rigshospitalet, University of Copenhagen, 2100 Copenhagen, Denmark; 5 Department of Oncology, Herlev and Gentofte Hospital, University of Copenhagen, 2730 Herlev, Denmark; 6 Department of Medicine, Herlev and Gentofte Hospital, University of Copenhagen, 2730 Herlev, Denmark; 7 Department of Clinical Medicine, Faculty of Health and Medical Sciences, University of Copenhagen, 2200 Copenhagen, Denmark

**Keywords:** TGF-β, LAP-TGF-β, TGF-β activation, plasma kallikrein, PDAC, tumor microenvironment, ECM remodeling, tumor fibrosis, serum biomarker

## Abstract

Pancreatic ductal adenocarcinoma (PDAC) is a hard-to-treat cancer due to the collagen-rich (fibrotic) and immune-suppressed microenvironment. A major driver of this phenomenon is transforming growth factor beta (TGF-β). TGF-β is produced in an inactive complex with a latency-associated protein (LAP) that can be cleaved by plasma kallikrein (PLK), hereby releasing active TGF-β. The aim of this study was to evaluate LAP cleaved by PLK as a non-invasive biomarker for PDAC and tumor fibrosis. An ELISA was developed for the quantification of PLK-cleaved LAP-TGF-β in the serum of 34 patients with PDAC (stage 1–4) and 20 healthy individuals. Biomarker levels were correlated with overall survival (OS) and compared to serum type III collagen (PRO-C3) and type VI collagen (PRO-C6) pro-peptides. PLK-cleaved LAP-TGF-β was higher in patients with PDAC compared to healthy individuals (*p* < 0.0001). High levels (>median) of PLK-cleaved LAP-TGF-β were associated with poor OS in patients with PDAC independent of age and stage (HR 2.57, 95% CI: 1.22–5.44, *p* = 0.0135). High levels of PLK-cleaved LAP-TGF-β were associated with high PRO-C3 and PRO-C6, indicating a relationship between the PLK-cleaved LAP-TGF-β fragment, TGF-β activity, and tumor fibrosis. If these preliminary results are validated, circulating PLK-cleaved LAP-TGF-β may be a biomarker for future clinical trials.

## 1. Introduction

Pancreatic ductal adenocarcinoma (PDAC) has a 5-year survival rate of less than 11%, and is expected to be one of the leading causes of cancer death in the next decade [1,2]. PDAC is a very stroma-rich tumor, as the stroma may account for more than 80% of the total tumor mass [3]. The dense stroma is primarily a consequence of tumor fibrosis, also known as desmoplasia, and consists of extracellular matrix (ECM) components, including various collagens, immune cells, endothelial cells, and cancer-associated fibroblasts (CAFs) [3,4,5]. CAFs play a major role in the formation and remodeling of the ECM, including an increase in type III and VI collagen synthesis, which have been shown to be related to short overall survival (OS) in patients with PDAC [6,7]. Under normal conditions, fibroblasts are in a quiescent state, but they can be activated and differentiated into CAFs by factors such as interleukin-1 (IL-1) or transforming growth factor beta (TGF-β). The latter of these is considered as the master regulator of fibrosis [8]. Important roles for TGF-β have been shown in fibrotic diseases such as kidney fibrosis, non-alcoholic steatohepatitis, and systemic sclerosis, suggesting potential applicability for other indications to measure TGF-β [9,10,11].

TGF-β is not only associated with pro-fibrotic signaling, but is also important for maintaining homeostasis in healthy tissue. It can also function as a tumor-suppresser in early-stage tumors by inducing autophagy, apoptosis, cell cycle arrest, and regulation of the immune response [8,12,13]. In PDAC, high tissue levels of TGF-β are related to tumor progression, invasion, immune evasion, desmoplasia, and poor OS [12,13]. Many cell types produce and/or store TGF-β, including platelets, cancer cells, macrophages, and other immune cells [12,14,15,16,17,18]. When TGF-β is synthesized, two precursor TGF-β polypeptides are dimerized with a disulfide bridge and subsequently cleaved by Furin. The resulting polypeptide associates with the latency-associated peptide (LAP) to form a small latent complex. Before secretion, the small complex is covalently linked to latent TGF-β binding protein (LTBP) to form a large latent TGF-β complex, which crosslinks to ECM proteins like fibronectin in the extracellular space [19,20]. To enable active TGF-β signaling, the TGF-β needs to be released from this complex. This can be achieved in many ways, including integrin interaction, trombospondin-1 binding, or protease-mediated cleavage. TGF-β activating proteases include matrix metalloproteinase (MMP) 2 and 9; plasmin, a disintegrin and metalloproteinase (ADAM) with thrombospondin type 1 motifs; and plasma kallikrein (PLK) [19,21,22,23,24,25].

**Figure 1:**
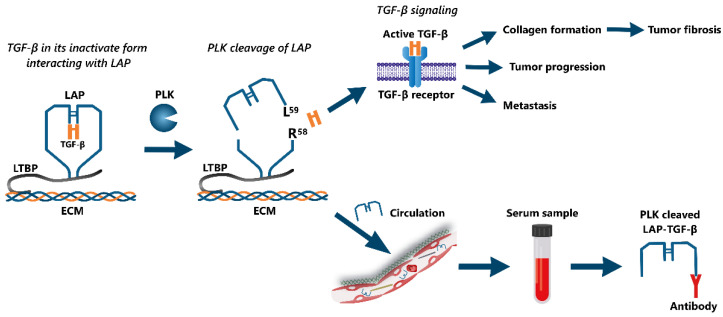
Illustration of plasma kallikrein (PLK)-mediated TGF-β activation: TGF-β in its latent state in complex with latency-associated peptide (LAP) (LAP-TGF-β) is bound to the extracellular matrix (ECM) via latent TGF-β binding protein (LTBP). PLK cleaves LAP between *R*^58^ and *L*^59^, resulting in the release of active TGF-β and the PLK-cleaved LAP-TGF-β fragment. The active TGF-β will induce multiple biological functions, including tumor progression, metastasis, and collagen formation, leading to tumor fibrosis. The PLK-cleaved LAP-TGF-β fragment is released into circulation, and as a result, becomes detectable in serum and plasma samples through antibody binding.

Active TGF-β has its primary function locally in the tissue, but may also end up in circulation as a non-invasive biomarker. A major challenge when measuring TGF-β in circulation is platelets in the samples, as these are a rich source of TGF-β that may be released during sample handling and cause interference [26,27]. TGF-β levels are generally measured in plasma and not serum, to avoid interference via the ex vivo activation of platelets [25,26,27,28,29]. Moreover, active TGF-β, once in circulation, is rapidly degraded with a half-life of less than 3 min [27,30]. To overcome these challenges, TGF-β activation could be indirectly measured by targeting degradation fragments that reflect TGF-β activation but have a longer half-life than the released TGF-β protein. In 2014, Hara et al. [24] identified the primary LAP cleavage site for plasma kallikrein (PLK), located between the R58 and L59 amino acid residues of the LAP-TGF-β complex (Figure 1). They successfully developed antibodies targeting the R58 and L59 cleavage sites, respectively, and were able to detect the R58 degradation product in tissue and measure the L59 degradation product in plasma from mice using immunohistochemistry and sandwich enzyme-linked immunosorbent assay (ELISA), respectively. The L59 degradation product had a half-life of ~8 h in plasma from mice and ~27 h in human plasma [24,25,31]. The R58 fragment has been detected in pancreatic stellate cells, a fibroblast-like cell type, and the L59 fragment in the supernatant from these cells, indicating a potential for PLK activated TGF-β as a biomarker for tumor fibrosis and TGF-β activity in patients with PDAC [31].

In this study, we developed and validated a competitive ELISA for the quantification of PLK-cleaved LAP-TGF-β in circulation (Figure 1). We evaluated the diagnostic and prognostic value for this biomarker in patients with PDAC, and investigated its association with circulating biomarkers of fibrosis.

## 2. Materials and Methods

### 2.1. Target Identification and Antibody Specificity

PLK-mediated cleavage of LAP-TGF-β at R’58 leads to a fragment of LAP being released into circulation [24]. The peptide ^59^LASPPSQGEV^68^ from the C-terminal side of the PLK cleavage site was used as a biomarker target for the LAP fragment, reflecting the PLK cleavage of TGF-β (Figure 1). BLAST was used on the amino acid sequence for homology to other human proteins, using NPS@: Network Protein Sequence Analysis with UniprotKB/Swiss-prot database, and no potential extracellular off-targets were found [32]. A conjugate of the peptide and Keyhole Limpet Hemocyanin (KLH), connected by a cysteine-linker (LASPPSQGEV-GGC-KLH), was used for the immunization of mice to produce monoclonal antibodies in the same manner as described in Nissen et al. [33,34].

**Figure 2:**
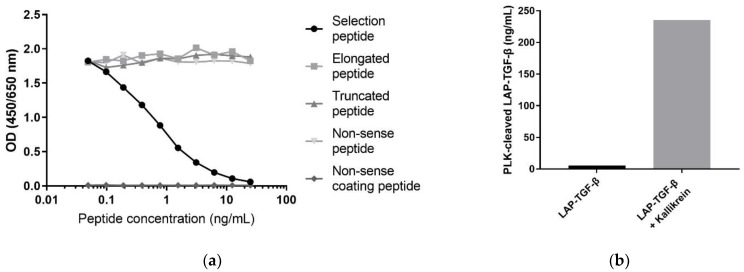
Assay specificity towards PLK-cleaved LAP-TGF-β: (**a**) Inhibition curve for the selection peptide (LASPPSQGEV), elongated peptide (RLASPPSQGEV), truncated peptide (ASPPSQGEV), non-sense selection peptide (PNASPLLGS), and a non-sense coater peptide (YPNASPLLGS-K-(Biotin)). The peptides were diluted in 2-fold dilution series starting from 25 ng/mL. (**b**) PLK-cleaved LAP-TGF-β measured after one hour incubation of LAP-TGF-β with or without kallikrein.

The specificity of the antibody towards the selection peptide (LASPPSQGEV) was evaluated using a preliminary competitive ELISA with biotinylated selection peptide (LASPPSQGEV-K-(biotin)) as a coater and a two-fold dilution series of selection peptide (LASPPSQGEV), elongated peptide (RLASPPSQGEV), truncated peptide (_ASPPSQGEV), and non-sense selection peptide (PNASPLLGS) (Figure 2a). In addition, a non-sense coater (YPNASPLLGS-K-(Biotin)) was also used, together with the selection peptide (LASPPSQGEV). To further evaluate the antibody specificity towards PLK-cleaved LAP-TGF-β, the epitope was measured via ELISA in samples of LAP-TGF-β incubated for one hour, with or without kallikrein (Figure 2b).

### 2.2. Assay Development and Validation

To evaluate the technical performance of the PLK-cleaved LAP-TGF-β ELISA, it underwent the following validation tests: determination of the measuring range, inter- and intra-assay variation, dilutional linearity, spiking accuracy, analyte stability, and interference tolerance of biotin, lipids, and hemoglobin.

The measurement range (lower limit of measurement range (LLMR) and upper limit of measurement range (ULMR)) and the inter- and intra-variation were estimated based on 10 independent runs of the assay using quality control samples comprising five human serum samples that span the linear part of the standard curve, three additional human serum samples, and two samples of known peptide concentration spiked into the assay buffer. LLMR and ULMR mark the boundaries of the linear range of the standard curve. Intra-assay variation was summarized as the mean coefficient of variance (CV%) between double-determinations of sample measurements of each assay run for the 10 quality control samples across the 10 independent runs. Inter-assay variation was summarized as the mean CV% between assay runs for the 10 quality controls samples across the 10 independent runs.

Dilutional linearity was tested by serially diluting four human serum samples 2-fold and calculating the percentage recovery relative to the undiluted samples.

Spiking accuracy was assessed by spiking a serum sample of high PLK-cleaved LAP-TGF-β concentrations into three human serum samples with low PLK-cleaved LAP-TGF-β concentrations and calculating the percentage recovery relative to the expected concentration in the spiked serum sample.

To determine the analyte stability, PLK-cleaved LAP-TGF-β was measured in three human serum samples after 24 h and 48 h of storage at 4 °C and 20 °C, with storage at −20 °C as a reference. Freeze/thaw stability was determined using PLK-cleaved LAP-TGF-β measurements of human serum samples after one to four freeze/thaw cycles with the samples after one cycle as a reference to calculate the percentage recovery.

As hemoglobin, biotin, and lipids are commonly interfering substances, the influence of these on the assay was investigated by spiking human serum samples with low or high concentrations of the substances (hemoglobin: low = 2.5 mg/mL, high = 5 mg/mL; biotin: low = 3 ng/mL, high = 9 ng/mL; and lipids: low = 1.5 mg/mL, high = 5 mg/mL). The interference was calculated as the percentage recovery of the spiked samples, with non-spiked samples as a reference.

### 2.3. PLK-Cleaved LAP-TGF-β Assay Protocol

The PLK-cleaved LAP-TGF-β assay went through optimization regarding the antibody/coater peptide ratio, assay buffer, incubation time, and temperature, as well as the conjugation of horseradish peroxidase (HRP) to the antibody. For the final protocol, 96-well streptavidin-coated plates were coated with 100 µL of 1.5 ng/mL biotinylated selection peptide (LASPPSQGEV-K-(biotin)) dissolved in assay buffer (25 mM TBS-BTB 2 g NaCl/L, pH 8.0), and incubated at 20 °C in darkness with 300 revolutions per minute (rpm), shaking for 30 min. The plates were washed five times in washing buffer (20 mmol/L TRIS, 50 mmol/L NaCl, pH 7.2), followed by the addition of 20 µL of a 2-fold serial dilution of selection peptide (LASPPSQGEV) starting at 38.75 ng/mL, 1:2 diluted serum sample or 1:4 diluted EDTA plasma sample to the wells, followed by the addition of 100 µL of 33.3 ng/mL HRP-conjugated PLK-cleaved LAP-TGF-β targeting antibody dissolved in assay buffer and incubated at 4 °C in darkness with 300 rpm shaking for 20 h (±1 h). After another five washes in washing buffer, 100 µL of tetramethylbenzidine (TMB) (Kem-En-Tec Diagnostics (Cat. No. 4380)) was added to each well, and the plates were incubated at 20 °C in darkness with 300 rpm shaking for 15 min, followed by the addition of 100 µL of 1% sulfuric acid to stop the reaction. Plates were analyzed using a VersaMax ELISA microplate reader (Molecular Devices, San Jose, CA, USA) at 450 nm, with 650 nm as reference. A standard curve was generated using a four-parametric mathematical fit, and the data were analyzed using GraphPad Prism (version 9).

### 2.4. Assessment of Type III and Type VI Collagen Formation

Formation of type III and type VI collagen was assessed by measuring the biomarkers PRO-C3 (cat. nr. 1700AF06) and PRO-C6 (cat. nr. 4000AF02), respectively, in human serum samples via ELISA [6,35,36]. The biomarkers were measured according to the manufacturer’s instructions (Nordic Bioscience A/S, Herlev, Denmark).

### 2.5. Subjects

The PLK-cleaved LAP-TGF-β biomarker was measured in pre-treatment serum samples from patients with PDAC (n = 34) and gender-matched healthy individuals (n = 20). Subject demographics are shown in Table 1, and include: age, gender, number of metastatic sites, body mass index (BMI), stage (American Joint Commission on cancer, 8th edition), and performance status [37,38]. In accordance with the Declaration of Helsinki, version 8, all subjects gave written informed consent. Healthy control samples (including the matched serum and plasma samples) were obtained from Valley BioMedical (Winchester, VA, USA), a commercial vendor with an appropriate institutional review board/independent ethical committee-approved sample collection. For the matched samples, both serum and plasma were obtained from six healthy individuals, also from Valley BioMedical (Winchester, VA, USA).

All patients with PDAC were included in the Danish BIOPAC study “BIOmarkers in patients with PAncreatic Cancer (BIOPAC)-can they provide new information of the disease and improve diagnosis and prognosis of the patients?” (ClinicalTrials.gov ID: NCT03311776;). The BIOPAC study is a prospective multicenter open cohort study with ongoing enrollment [38]. The patients were recruited from Herlev Hospital and Rigshospitalet from August 2015 until September 2018.

The BIOPAC study protocol was approved by the Danish Ethics Committee (VEK KA-20060113 and the retrospective protocol VEK H-17039022) and the Danish Data Protection Agency (j.nr. 2006-41-6848, 2012-58-0004; HGH-2015-027; I-Suite j.nr. 03960; and PACTICUS P-2020-834). The included patients had histologically confirmed PDAC and received treatment according to the national guidelines. Samples were obtained before the first treatment (surgery or first-line palliative chemotherapy), and clinical data were collected from patients prospectively. The serum samples were measured blinded to the clinical information. The patients were followed until May 2022 or death, whichever came first.

### 2.6. Statistics

Biomarker levels in healthy individuals and patients with PDAC or subgroups of patients were compared using the Mann-Whitney test. Wilson/Brown Receiver operating characteristic (ROC) curve analysis was used to further investigate the diagnostic potential of the biomarker. Correlations between PLK-cleaved LAP-TGF-β levels in matched serum and plasma samples were calculated using Pearson correlation. Kaplan-Meier survival analysis was used to assess differences in OS between high (>median) and low (<median) PLK-cleaved LAP-TGF-β biomarker levels in patients with PDAC. The prognostic value of the PLK-cleaved LAP-TGF-β biomarker was further evaluated using univariate and multivariate Cox proportional-hazards regression models, including PLK-cleaved LAP-TGF-β levels (dichotomized at the median), age (continuous scale), and stage (stage 1–3 versus stage 4).

Statistical analyses and graphic designs were made using GraphPad Prism (version 9) and MedCalc (version 19.3) software.

## 3. Results

### 3.1. Technical Evaluation of the PLK-Cleaved LAP-TGF-β ELISA

The measurement range (LLMR to ULMR) of the PLK-cleaved LAP-TGF-β assay was determined to be 0.05 to 2.2 ng/mL, with an IC50 of 0.34 ng/mL. The intra- and inter-assay variations were 3% and 11%, respectively. The mean dilution recovery for human serum was 115%, 114%, and 101%, observed from undiluted to a 1:2, 1:4, and 1:8 dilution, respectively, and the mean spiking recovery was 94%. After five freeze/thaw cycles, the analyte recovery in serum was 113%. Analyte recovery from human serum after storage at 4 °C for 48 h was from 98%, demonstrating a much higher stability of the PLK-cleaved LAP-TGF-β fragment than previously shown [31]. For storage at 20 °C for 24 h, the recovery was 88%. Analyte recoveries for the interference of hemoglobin, biotin, and lipids were 101%, 102%, and 98% for low concentrations, and 100%, 105%, and 100% for high concentrations, respectively. Altogether, this demonstrates that the assay is technically stable, robust, and unaffected by the most commonly interfering substances from serum. The technical evaluation results are summaries in Table 2.

### 3.2. Specificity of the PLK-Cleaved LAP-TGF-β ELISA

To determine the antibody specificity of the PLK-cleaved LAP-TGF-β ELISA, the signal inhibition of the selection peptide (LASPPSQGEV), elongated peptide (RLASPPSQGEV), truncated peptide (ASPPSQGEV), non-sense standard peptide (PNASPLLGS), and a non-sense coater peptide (YPNASPLLGS-K-(Biotin)) were used, as shown in Figure 2a. The selection peptide led to a dose-dependent signal inhibition, while the truncated, elongated, and non-sense peptide showed no signal inhibition, and no signal was detected with the non-sense coating peptide. This demonstrates a high specificity of the assay towards the PLK-cleaved LAP-TGF-β neoepitope (LASPPSQGEV).

To further support the specificity and to confirm the ability to target PLK-cleaved LAP-TGF-β, a cleavage experiment was performed. The LAP-TGF-β antibody only showed reactivity towards kallikrein-cleaved LAP-TGF-β, and not intact LAP-TGF-β (Figure 2b).

### 3.3. PLK-Cleaved LAP-TGF-β Levels Were Correlated in Matched Serum and Plasma Samples

Platelet derived TGF-β could result in higher LAP-TGF-β levels in serum compared to plasma [26,27,29,39]. To evaluate this interference, PLK-cleaved LAP-TGF-β was measured in matched serum and citrate-plasma samples. Citrate tubes were used, as these have been shown to result in lower TGF-β levels than EDTA tubes [26].

**Figure 3:**
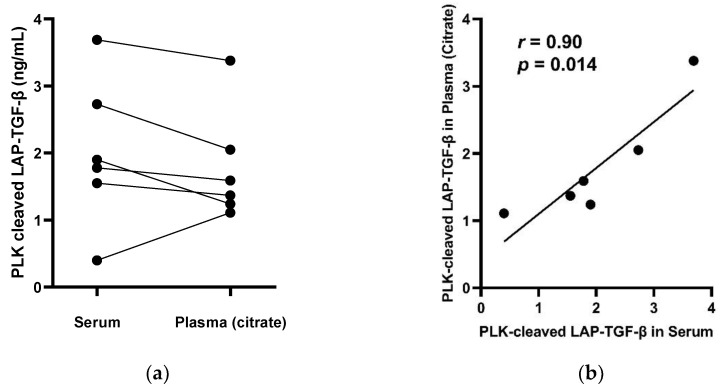
Biomarker levels of PLK-cleaved LAP-TGF-β in matched serum and citrate plasma samples: (**a**) Matched samples are connected by lines. (**b**) Correlation between the serum and plasma PLK-cleaved LAP-TGF-β levels shown in (a) was tested with Pearson test; Pearson’s *r* (*r*) and *p*-values are shown. The line represents simple linear regression.

We did not observe a notable difference in PLK-cleaved LAP-TGF-β levels between serum and plasma samples from the same donors (Figure 3a). A significant correlation between PLK-cleaved LAP-TGF-β measured in serum and plasma was found using Pearson correlation (Pearson’s *r* = 0.90 and *p* = 0.014) (Figure 3b).

### 3.4. PLK-Cleaved LAP-TGF-β Is Elevated in Serum from Patients with PDAC, and Shows Diagnostic Potential

**Figure 4:**
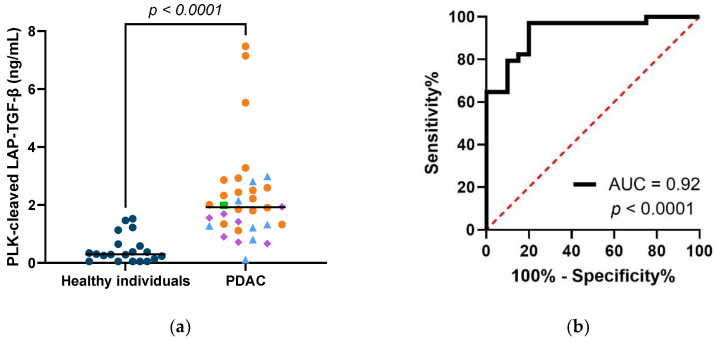
Diagnostic potential of PLK-cleaved LAP-TGF-β: (**a**) Serum levels of PLK-cleaved LAP-TGF-β in healthy individuals (n = 20) and patients with PDAC (n = 34). Black lines indicate median values. Patients in stages 1, 2, 3, and 4 have shape and color codes being green square, purple diamonds, light blue triangles, and orange circles, respectively. (**b**) Receiver operating characteristic curve for the patients and healthy individual mentioned in (a), area under curve (AUC).

To evaluate the clinical and biological relevance of the PLK-cleaved LAP-TGF-β biomarker, it was measured in pretreatment serum samples from 34 patients with PDAC (stage 1 (n = 1), stage 2 (n = 7), stage 3 (n = 8), and stage 4 (n = 18)), and healthy individuals (n = 20). The clinical characteristics are shown in Table 1. The concentration of PLK-cleaved LAP-TGF-β was significantly elevated (*p* < 0.0001) in patients with PDAC (median = 1.92 ng/mL, range 0.11–7.48) compared to healthy individuals (median = 0.30 ng/mL, range: 0.05–1.52) (Figure 4a). In this preliminary study, the biomarker was able to significantly discriminate between healthy individuals and patients with PDAC, with an area under the receiver operating characteristic (AUROC) curve of 0.92, *p* < 0.0001 (Figure 4b), further supporting the diagnostic potential of PLK-cleaved LAP-TGF-β.

### 3.5. Overall Survival in Patients with PDAC Is Associated with PLK-Cleaved LAP-TGF-β

**Figure 5:**
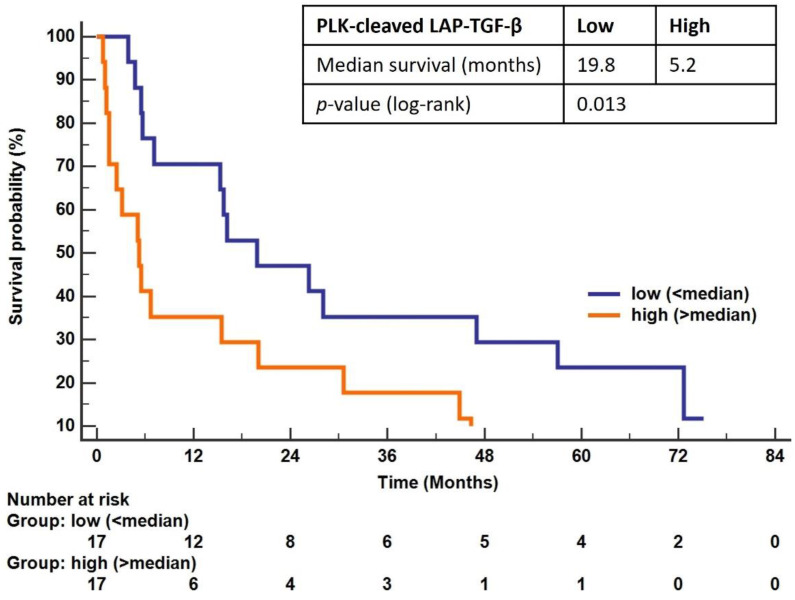
Prognostic properties of PLK-cleaved LAP-TGF-β: Kaplan-Meier survival curves showing the association between serum PLK-cleaved LAP-TGF-β levels (dichotomized into low (<median (blue curve)) and high (>median (orange curve)), and overall survival for patients with PDAC. Median survival and log-rank test are shown.

The prognostic value of PLK-cleaved LAP-TGF-β was evaluated in the patients with PDAC after dividing the patients into two groups based on PLK-cleaved LAP-TGF-β levels above and below the median, and examining using Kaplan-Meier analysis and uni- and multivariate Cox proportional hazards models for an association with OS. The median OS was 5.2 months (95% CI: 1.5–60.2) in patients with high levels of PLK-cleaved LAP-TGF-β (above median), compared to 19.8 months (95% CI: 5.6–57.1) for patients with low levels of PLK-cleaved LAP-TGF-β (below median). Likewise, when evaluated through univariate analysis, patients with high levels of PLK-cleaved LAP-TGF-β had a significantly decreased OS compared to patients with low levels (log-rank *p* = 0.013) (Figure 5).

The association between high levels of PLK-cleaved LAP-TGF-β and poor OS was statistically significant after adjusting for age and the subdivision of metastatic and non-metastatic tumors (stage 1–3 versus stage 4): (HR: 2.57, 95% CI: 1.22–5.44, *p* = 0.014).

### 3.6. High PLK-Cleaved LAP-TGF-β Levels Associate with Both Collagen Type III and Type VI Formation in Patients with PDAC

**Figure 6:**
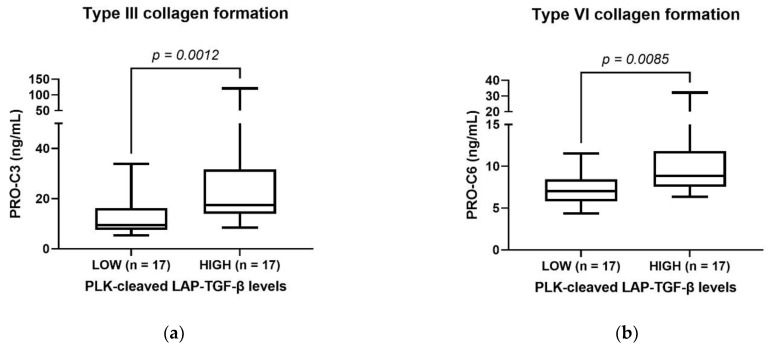
PLK-cleaved LAP-TGF-β levels associate with collagen formation: Biomarker levels of PRO-C3 (**a**) and PRO-C6 (**b**) from patients with PDAC dichotomized into low PLK-cleaved LAP-TGF-β levels (<median, n = 17) and high PLK-cleaved LAP-TGF-β levels (>median, n = 17). The groups were compared using Mann-Whitney test; *p*-value is shown.

To evaluate the association between collagen formation (fibrosis) and PLK-cleaved LAP-TGF-β, pre-treatment serum levels of type III collagen (PRO-C3) and type VI collagen (PRO-C6), pro-peptides were measured in patients with PDAC. Both PRO-C3 and PRO-C6 were significantly higher in patients with high levels of PLK-cleaved LAP-TGF-β (>median), compared to patients with low levels of PLK-cleaved LAP-TGF-β (<median) (Figure 6). This indicates that PLK-activated TGF-β is associated with increased collagen type III and VI formation in patients with PDAC.

## 4. Discussion

In this study, we developed and validated an ELISA assay for measuring PLK-activated TGF-β by targeting the cleaved LAP-TGF-β fragment in serum. Analyte stability and ex vivo activated platelet derived TGF-β are two major issues when attempting to measure TGF-β in serum directly. Large amounts of TGF-β can be released ex vivo from platelets, resulting in false-positive measurements of TGF-β [17,26,39,40]. To prevent the ex vivo activation of platelet derived TGF-β, TGF-β is often measured in plasma instead of serum [26,27]. With the PLK-cleaved LAP-TGF-β assay, we found a similarity and correlation between the PLK-cleaved LAP-TGF-β levels in matched serum and plasma samples, which indicated that the analyte was not influenced by ex vivo platelet derived activation in serum.

We demonstrated the diagnostic and prognostic potential of PLK-cleaved LAP-TGF-β in a preliminary study, with serum samples from 34 patients with PDAC and 20 healthy individuals. We showed that PLK-cleaved LAP-TGF-β was significantly higher in serum from patients with PDAC, compared to healthy individuals. To our knowledge, previously published studies only investigated this fragment in plasma from patients with liver diseases, where it was found to be a potential marker for monitoring the clinical course of chronic liver disease [25,28]. In the present study, high levels of PLK-cleaved LAP-TGF-β (>median) were significantly associated with short OS in patients with PDAC, and independent of age and stage. We also found that high levels of PLK-cleaved LAP-TGF-β were associated with type III and type VI collagen formation, suggesting an association with desmoplasia in PDAC. Both type III and type VI collagen formation have been shown to be associated with other common markers of fibrosis, including α-smooth muscle actin (α-SMA), and they are known to be increased and predictive of poor OS in patients with PDAC [6,37,41,42,43].

TGF-β has earlier been shown to be elevated in serum from patients with pancreatic cancer, as well as being predictive of poor OS [44,45]. Elevated circulating TGF-β levels have also been shown to predict poor OS in other types of cancers, including pulmonary cancer, hepatic cancer, and leukemia [46,47,48]. These studies measured TGF-β in serum using an ELISA with an antibody that targets either activated TGF-β or the LAP-TGF-β complex directly. These assays will either have the previously discussed issues of analyte stability or will not be able to separate active TGF-β from latent TGF-β which could influence the results, depending on which form of TGF-β the antibody is targeting.

There are and have been many ongoing clinical trials investigating the utility of various anti-TGF-β drugs for use in cancer treatment; however, despite promising preclinical studies, many of these trials fail due to lack of consistency or being unable to recapitulate the data shown in preclinical studies [49,50,51,52]. This demonstrates an ongoing need for better translational TGF-β biomarkers. The PLK-cleaved LAP-TGF-β ELISA cross-reacts to mice, and could be used in pre-clinical studies as well. Thus, the PLK-cleaved LAP-TGF-β biomarker could potentially be used for identifying patients that would benefit the most, and for monitoring the effect of anti-TGF-β drugs in clinical as well as pre-clinical studies.

The present study has several limitations. Since the number of patients with PDAC and healthy individuals are low, future validation in larger cohorts are needed to determine the utility of this biomarker in patients with PDAC. In addition, the group of healthy individuals were slightly younger than the patient group, which can affect the diagnostic potential shown in this study. Furthermore, the PDAC patients included in the BIOPAC study have good performance status, and are more likely to receive anticancer therapy, which may provide selection bias [37]. Another limitation is that the assay only measures the activation of TGF-β that is activated through the PLK-mediated cleavage of LAP. Although we demonstrated the applicability of PLK activated TGF-β in PDAC, TGF-β can also be released from the LAP complex, due to other factors including LAP cleavage mediated by other proteases and integrin interactions [19,21,22,23].

## 5. Conclusions

In summary, we developed a competitive ELISA for the measurement of PLK-activated TGF-β with a high analyte stability and indications of being unaffected by ex vivo platelet derived TGF-β activation. We demonstrated the diagnostic and prognostic value of this biomarker in patients with PDAC. Furthermore, we showed an association between high levels of PLK activated TGF-β, and type III and type VI collagen formation in patients with PDAC, suggesting the involvement of TGF-β in the induction of tumor fibrosis. These are preliminary results, but if validated, circulating PLK-cleaved LAP-TGF-β may be a biomarker for future clinical trials in relation to anti-TGF beta drugs, as well as tumor fibrosis modulation.

## Figures and Tables

**Table 1:** Patient demographics and clinical profile for included patients with PDAC, and healthy individuals.

Clinical Variables	Patients with PDAC (n = 39)	Healthy Individuals (n = 20)
Age (years), Median (min–max)	69 (52–79)	58 (45–72)
Gender, n (%) Male Female	17 (50%) 17 (50%)	10 (50%) 10 (50%)
Number of metastatic sites, n (%) 0 site ≥1 site	16 (47%) 18 (53%)	
Body mass index (BMI), Median (min-max)	23 (16–31)	
Stage 1 2 3 4	1 (3%) 7 (21%) 8 (24%) 18 (53%)	
Performance status, n (%) 0 1 2 Unknown	13 (38%) 13 (38%) 3 (9%) 5 (15%)	

**Table 2:** Technical validation summary.

Test	Results
IC50	0.34 ng/mL
Measurement range (LLMR-ULMR)	0.05–2.2 ng/mL
Detection range (LLOD-ULOD)	0.02–10.8 ng/mL
Dilution recovery (1:2, 1:4, 1:8)	115%, 114%, 101%
Spiking recovery (serum in serum)	94%
Interference (Hemoglobin, low/high conc)	101%/100%
Interference (biotin, low/high conc)	102%/105%
Interference (lipids, low/high conc)	98%/100%
Inter-assay variation	11%
Intra-assay variation	3%
Analyte stability (48 h 4 °C/24 h 20 °C)	98%/88%
Freeze/thaw stability up to four cycles	113%

## Data Availability

Original biomarker data can be obtained upon reasonable request.

## References

[1] Siegel R.L., Miller K.D., Fuchs H.E., Jemal A. (2022). Cancer Statistics, 2022. CA. Cancer J. Clin..

[2] Mattiuzzi C., Lippi G. (2019). Current Cancer Epidemiology. J. Epidemiol. Glob. Health.

[3] Bulle A., Lim K.H. (2020). Beyond Just a Tight Fortress: Contribution of Stroma to Epithelial-Mesenchymal Transition in Pancreatic Cancer. Signal Transduct. Target. Ther..

[4] Willumsen N., Jensen C., Green G., Nissen N.I., Neely J., Nelson D.M., Pedersen R.S., Frederiksen P., Chen I.M., Boisen M.K. (2022). Fibrotic Activity Quantified in Serum by Measurements of Type III Collagen Pro-Peptides Can Be Used for Prognosis across Different Solid Tumor Types. Cell. Mol. Life Sci..

[5] Feig C., Gopinathan A., Neesse A., Chan D.S., Cook N., Tuveson D. (2012). a The Pancreas Cancer Microenvironment. Clin. Cancer Res..

[6] Nissen N.I., Johansen A.Z., Chen I., Johansen J.S., Pedersen R.S., Hansen C.P., Karsdal M.A., Willumsen N. (2022). Collagen Biomarkers Quantify Fibroblast Activity In Vitro and Predict Survival in Patients with Pancreatic Ductal Adenocarcinoma. Cancers.

[7] Chen X., Song E. (2019). Turning Foes to Friends: Targeting Cancer-Associated Fibroblasts. Nat. Rev. Drug Discov..

[8] Meng X., Nikolic-Paterson D.J., Lan H.Y. (2016). TGF-β: The Master Regulator of Fibrosis. Nat. Rev. Nephrol..

[9] Rice L.M., Padilla C.M., McLaughlin S.R., Mathes A., Ziemek J., Goummih S., Nakerakanti S., York M., Farina G., Whitfield M.L. (2015). Fresolimumab Treatment Decreases Biomarkers and Improves Clinical Symptoms in Systemic Sclerosis Patients. J. Clin. Investig..

[10] Schwabe R.F., Tabas I., Pajvani U.B. (2020). Mechanisms of Fibrosis Development in Nonalcoholic Steatohepatitis. Gastroenterology.

[11] Yamamoto T., Noble N.A., Miller D.E., Border W.A. (1994). Sustained Expression of TGF-Β1 Underlies Development of Progressive Kidney Fibrosis. Kidney Int..

[12] Principe D.R., Timbers K.E., Atia L.G., Koch R.M., Rana A. (2021). TGFβ Signaling in the Pancreatic Tumor Microenvironment. Cancers.

[13] Seoane J., Gomis R.R. (2017). TGF-β Family Signaling in Tumor Suppression and Cancer Progression. Cold Spring Harb. Perspect. Biol..

[14] Ueshima E., Fujimori M., Kodama H., Felsen D., Chen J., Durack J.C., Solomon S.B., Coleman J.A., Srimathveeravalli G. (2019). Macrophage-Secreted TGF-β 1 Contributes to Fibroblast Activation and Ureteral Stricture after Ablation Injury. Am. J. Physiol. Physiol..

[15] Acerbi I., Cassereau L., Dean I., Shi Q., Au A., Park C., Chen Y.Y., Liphardt J., Hwang E.S., Weaver V.M. (2015). Human Breast Cancer Invasion and Aggression Correlates with ECM Stiffening and Immune Cell Infiltration. Integr. Biol..

[16] Nagaraj N.S., Datta P.K. (2010). Targeting the Transforming Growth Factor-β Signaling Pathway in Human Cancer. Expert Opin. Investig. Drugs.

[17] Assoian R.K., Komoriya A., Meyers C.A., Miller D.M., Sporn M.B. (1983). Transforming Growth Factor-Beta in Human Platelets. Identification of a Major Storage Site, Purification, and Characterization. J. Biol. Chem..

[18] Liu M., Fu X., Jiang L., Ma J., Zheng X., Wang S., Guo H., Tian T., Nan K., Wang W. (2021). Colon Cancer Cells Secreted CXCL11 via RBP-Jκ to Facilitated Tumour-associated Macrophage-induced Cancer Metastasis. J. Cell. Mol. Med..

[19] Hayashi H., Sakai T. (2012). Biological Significance of Local TGF-β Activation in Liver Diseases. Front. Physiol..

[20] Dallas S.L., Sivakumar P., Jones C.J.P., Chen Q., Peters D.M., Mosher D.F., Humphries M.J., Kielty C.M. (2005). Fibronectin Regulates Latent Transforming Growth Factor-β (TGFβ) by Controlling Matrix Assembly of Latent TGFβ-Binding Protein-1. J. Biol. Chem..

[21] Lyons R.M., Gentry L.E., Purchio A.F., Moses H.L. (1990). Mechanism of Activation of Latent Recombinant Transforming Growth Factor Beta 1 by Plasmin. J. Cell Biol..

[22] Yu Q., Stamenkovic I. (2000). Cell Surface-Localized Matrix Metalloproteinase-9 Proteolytically Activates TGF-β and Promotes Tumor Invasion and Angiogenesis. Genes Dev..

[23] Bourd-Boittin K., Bonnier D., Leyme A., Mari B., Tuffery P., Samson M., Ezan F., Baffet G., Theret N. (2011). Protease Profiling of Liver Fibrosis Reveals the ADAM Metallopeptidase with Thrombospondin Type 1 Motif, 1 as a Central Activator of Transforming Growth Factor Beta. Hepatology.

[24] Hara M., Kirita A., Kondo W., Matsuura T., Nagatsuma K., Dohmae N., Ogawa S., Imajoh-Ohmi S., Friedman S.L., Rifkin D.B. (2014). LAP Degradation Product Reflects Plasma Kallikrein-Dependent TGF-β Activation in Patients with Hepatic Fibrosis. Springerplus.

[25] Hara M., Inoue I., Yamazaki Y., Kirita A., Matsuura T., Friedman S.L., Rifkin D.B., Kojima S. (2015). L59 TGF-β LAP Degradation Products Serve as a Promising Blood Biomarker for Liver Fibrogenesis in Mice. Fibrogenesis Tissue Repair.

[26] O’Brien P.J., Ramanathan R., Yingling J.M., Baselga J., Rothenberg M.L., Carducci M., Daly T., Adcock D., Lahn M. (2008). Analysis and Variability of TGFbeta Measurements in Cancer Patients with Skeletal Metastases. Biologics.

[27] Mancini D., Monteagudo J., Suárez-Fariñas M., Bander J., Varshney R., Gonzalez J., Coller B.S., Ahamed J. (2018). New Methodologies to Accurately Assess Circulating Active Transforming Growth Factor-Β1 Levels: Implications for Evaluating Heart Failure and the Impact of Left Ventricular Assist Devices. Transl. Res..

[28] Yokoyama H., Masaki T., Inoue I., Nakamura M., Mezaki Y., Saeki C., Oikawa T., Saruta M., Takahashi H., Ikegami M. (2019). Histological and Biochemical Evaluation of Transforming Growth Factor-β Activation and Its Clinical Significance in Patients with Chronic Liver Disease. Heliyon.

[29] Kropf J., Schurek J.O., Wollner A., Gressner A.M. (1997). Immunological Measurement of Transforming Growth Factor-Beta I (TGF- Β1) in Blood; Assay Development and Comparison. Clin. Chem..

[30] Coffey R.J., Kost L.J., Lyons R.M., Moses H.L., LaRusso N.F. (1987). Hepatic Processing of Transforming Growth Factor Beta in the Rat. Uptake, Metabolism, and Biliary Excretion. J. Clin. Investig..

[31] Teraoka R., Hara M., Kikuta K., Hirooka Y., Furutani Y., Shimosegawa T., Masamune A., Kojima S. (2017). Plasma Kallikrein-Dependent Transforming Growth Factor-β Activation in Patients With Chronic Pancreatitis and Pancreatic Cancer. Pancreas.

[32] Combet C., Blanchet C., Geourjon C., Deléage G. (2000). NPS@: Network Protein Sequence Analysis. Trends Biochem. Sci..

[33] Nissen N.I., Kehlet S., Johansen A.Z., Chen I.M., Karsdal M., Johansen J.S., Diab H.M.H., Jørgensen L.N., Sun S., Manon-Jensen T. (2021). Noninvasive Prognostic Biomarker Potential of Quantifying the Propeptides of Type XI Collagen Alpha-1 Chain (PRO-C11) in Patients with Pancreatic Ductal Adenocarcinoma. Int. J. Cancer.

[34] Gefter M.L., Margulies D.H., Scharff M.D. (1977). A Simple Method for Polyethylene Glycol-Promoted Hybridization of Mouse Myeloma Cells. Somatic Cell Genet..

[35] Nielsen M.J., Nedergaard A.F., Sun S., Veidal S.S., Larsen L., Zheng Q., Suetta C., Henriksen K., Christiansen C., Karsdal M.A. (2013). The Neo-Epitope Specific PRO-C3 ELISA Measures True Formation of Type III Collagen Associated with Liver and Muscle Parameters. Am. J. Transl. Res..

[36] Sun S., Henriksen K., Karsdal M.A., Byrjalsen I., Rittweger J., Armbrecht G., Belavy D.L., Felsenberg D., Nedergaard A.F. (2015). Collagen Type III and VI Turnover in Response to Long-Term Immobilization. PLoS ONE.

[37] Chen I.M., Willumsen N., Dehlendorff C., Johansen A.Z., Jensen B.V., Hansen C.P., Hasselby J.P., Bojesen S.E., Pfeiffer P., Nielsen S.E. (2020). Clinical Value of Serum Hyaluronan and Propeptide of Type III Collagen in Patients with Pancreatic Cancer. Int. J. Cancer.

[38] Chen I., Jensen B.V., Bojesen S.E., Johansen A.Z., Schultz N.A., Hansen C.P., Hasselby J.P., Holl N.H., Nissen M.H.B., Bjerregaard J.K. (2019). Identification of New Biomarkers in Patients with Pancreatic Cancer (BIOPAC): A Study Protocol of an Open Cohort Study. Cancer Sci. Ther..

[39] Meyer A., Wang W., Qu J., Croft L., Degen J.L., Coller B.S., Ahamed J. (2012). Platelet TGF-Β1 Contributions to Plasma TGF-Β1, Cardiac Fibrosis, and Systolic Dysfunction in a Mouse Model of Pressure Overload. Blood.

[40] Grainger D.J., Mosedale D.E., Metcalfe J.C., Weissberg P.L., Kemp P.R. (1995). Active and Acid-Activatable TGF-β in Human Sera, Platelets and Plasma. Clin. Chim. Acta.

[41] Willumsen N., Bager C., Karsdal M.A. (2019). Matrix Metalloprotease Generated Fragments of Type VI Collagen Have Serum Biomarker Potential in Cancer–A Proof of Concept Study. Transl. Oncol..

[42] Kerbert A.J.C., Gupta S., Alabsawy E., Dobler I., Lønsmann I., Hall A., Nielsen S.H., Nielsen M.J., Gronbaek H., Amoros À. (2021). Biomarkers of Extracellular Matrix Formation Are Associated with Acute-on-Chronic Liver Failure. JHEP Reports.

[43] Rønnow S.R., Dabbagh R.Q., Genovese F., Nanthakumar C.B., Barrett V.J., Good R.B., Brockbank S., Cruwys S., Jessen H., Sorensen G.L. (2020). Prolonged Scar-in-a-Jar: An in Vitro Screening Tool for Anti-Fibrotic Therapies Using Biomarkers of Extracellular Matrix Synthesis. Respir. Res..

[44] Zhao J., Liang Y., Yin Q., Liu S., Wang Q., Tang Y., Cao C. (2016). Clinical and Prognostic Significance of Serum Transforming Growth Factor-Beta1 Levels in Patients with Pancreatic Ductal Adenocarcinoma. Braz. J. Med. Biol. Res..

[45] Park H., Bang J., Nam A., Park J.E., Jin M.H., Bang Y., Oh D. (2020). The Prognostic Role of Soluble TGF-beta and Its Dynamics in Unresectable Pancreatic Cancer Treated with Chemotherapy. Cancer Med..

[46] Li J., Shen C., Wang X., Lai Y., Zhou K., Li P., Liu L., Che G. (2019). Prognostic Value of TGF-β in Lung Cancer: Systematic Review and Meta-Analysis. BMC Cancer.

[47] Lin T.-H., Shao Y.-Y., Chan S.-Y., Huang C.-Y., Hsu C.-H., Cheng A.-L. (2015). High Serum Transforming Growth Factor-Β1 Levels Predict Outcome in Hepatocellular Carcinoma Patients Treated with Sorafenib. Clin. Cancer Res..

[48] Rouce R.H., Shaim H., Sekine T., Weber G., Ballard B., Ku S., Barese C., Murali V., Wu M.-F., Liu H. (2016). The TGF-β/SMAD Pathway Is an Important Mechanism for NK Cell Immune Evasion in Childhood B-Acute Lymphoblastic Leukemia. Leukemia.

[49] Teixeira A.F., ten Dijke P., Zhu H.-J. (2020). On-Target Anti-TGF-β Therapies Are Not Succeeding in Clinical Cancer Treatments: What Are Remaining Challenges?. Front. Cell Dev. Biol..

[50] Ciardiello D., Elez E., Tabernero J., Seoane J. (2020). Clinical Development of Therapies Targeting TGFβ: Current Knowledge and Future Perspectives. Ann. Oncol..

[51] Kim B.-G., Malek E., Choi S.H., Ignatz-Hoover J.J., Driscoll J.J. (2021). Novel Therapies Emerging in Oncology to Target the TGF-β Pathway. J. Hematol. Oncol..

[52] Huynh L., Hipolito C., ten Dijke P. (2019). A Perspective on the Development of TGF-β Inhibitors for Cancer Treatment. Biomolecules.

